# A Pragmatic Approach to Adverse Outcome Pathway Development and Evaluation

**DOI:** 10.1093/toxsci/kfab113

**Published:** 2021-09-17

**Authors:** Terje Svingen, Daniel L Villeneuve, Dries Knapen, Eleftheria Maria Panagiotou, Monica Kam Draskau, Pauliina Damdimopoulou, Jason M O’Brien

**Affiliations:** 1 Division of Diet, Disease Prevention and Toxicology, National Food Institute, Technical University of Denmark, Lyngby DK 2800, Denmark; 2 Great Lakes Toxicology and Ecology Division, United States Environmental Protection Agency, Duluth, Minnesota 55804, USA; 3 Zebrafishlab, Department of Veterinary Sciences, University of Antwerp, Wilrijk 2610, Belgium; 4 Department of Clinical Science, Intervention and Technology, Karolinska Institutet and Karolinska University Hospital, Stockholm 14186, Sweden; 5 Ecotoxicology and Wildlife Health Division, Environment and Climate Change Canada, Ottawa, Ontario K1S 5B6, Canada

**Keywords:** risk assessment, chemical regulation, AOP-KB, AOP-wiki, toxicology

## Abstract

The adverse outcome pathway (AOP) framework provides a practical means for organizing scientific knowledge that can be used to infer cause-effect relationships between stressor events and toxicity outcomes in intact organisms. It has reached wide acceptance as a tool to aid chemical safety assessment and regulatory toxicology by supporting a systematic way of predicting adverse health outcomes based on accumulated mechanistic knowledge. A major challenge for broader application of the AOP concept in regulatory toxicology, however, has been developing robust AOPs to a level where they are peer reviewed and accepted. This is because the amount of work required to substantiate the modular units of a complete AOP is considerable, to the point where it can take years from start to finish. To help alleviate this bottleneck, we propose a more pragmatic approach to AOP development whereby the focus becomes on smaller blocks. First, we argue that the key event relationship (KER) should be formally recognized as the core building block of knowledge assembly within the AOP knowledge base (AOP-KB), albeit framing them within full AOPs to ensure regulatory utility. Second, we argue that KERs should be developed using systematic review approaches, but only in cases where the underlying concept does not build on what is considered canonical knowledge. In cases where knowledge is considered canonical, rigorous systematic review approaches should not be required. It is our hope that these approaches will contribute to increasing the pace at which the AOP-KB is populated with AOPs with utility for chemical safety assessors and regulators.

To meet the needs for chemical safety assessment in the 21st century, regulatory toxicology aims to increase the capacity to assess the ever-growing number of chemical substances and, at the same time, reduce its reliance on animal testing. This requires development and adoption of test methods capable of predicting probable adverse health effects in intact organisms, including humans. One of the major challenges with this is the lack of sufficient understanding of the complex mechanisms taking place in response to stressors such as chemical substances in order to account for the broad palette of potential human and environmental health outcomes. The adverse outcome pathway (AOP) concept, introduced over a decade ago (Ankley *et al.*, 2010), has evolved into a promising framework for assembling scientific knowledge and evidence to aid in establishing new approaches that can adequately predict *in vivo* effect outcomes.

There is a flurry of AOP activities currently taking place across toxicological disciplines and the framework has become integral in several larger research programs funded by, for instance, the European Commission under Horizon 2020 such as the EURION cluster (Street *et al.*, 2021). Despite this growing interest, the AOP knowledge base (AOP-KB), which serves as the globally accessible repository for AOPs developed in accordance with the principles and guidance established by the Organisation for Economic Co-operation and Development (OECD)-AOP program (https://www.oecd.org/chemicalsafety/testing/adverse-outcome-pathways-molecular-screening-and-toxicogenomics.htm; last accessed September 10, 2021), contains just 17 endorsed AOPs at the time of writing this article (August 2021); with perhaps another dozen AOPs in a scientific review. The remaining 350 entries found in the AOP-wiki (www.aopwiki.org; last accessed September 10, 2021 ), the most commonly used interface for accessing and developing AOPs, are under development. Many of these AOPs consist of little more than a series of event titles without significant assembly of supporting knowledge and evidence. A major reason for this slow development of the AOP-KB is the substantial amount of work required to develop and review an AOP to a level where it can be endorsed by the OECD (https://doi.org/10.1787/2415170X; last accessed September 10, 2021). This, alongside the lack of commensurate professional recognition of the scholarly effort invested in rigorous AOP development, is a major hurdle that prevents many potential AOP developers from either embarking on the task all together, or saps motivation once it becomes clear how much work is required. These points are also reflected in the discrepancy between “purely conceptual” or “putative” AOPs that have been published in the open literature, eg (Barenys *et al.*, 2020; Franssen *et al.*, 2021; Johansson *et al.*, 2020; Martinovic-Weigelt *et al.*, 2017; Palermo *et al.*, 2021; Vinken, 2020; Weiss *et al.*, 2021; Yang *et al.*, 2019), versus those that exist and are under active development in the AOP-wiki .

It is not a grueling task to provide a conceptual idea for an AOP nor to provide some basic level of background information. Taking the next steps to elaborate the entire sequence of events, however, particularly with respect to the weight-of-evidence documentation and evaluation as required under the OECD-AOP development guidelines (OECD, 2018), can be a major undertaking . To promote the development of new AOPs and to increase the pace at which the AOP-wiki can be populated with scientifically reviewed and endorsed AOPs, we suggest a more pragmatic approach to AOP development where more focus is put on smaller units of development and review. The end goal of this approach remains developing complete AOPs, but will allow for more speedy development as full AOPs in essence can evolve from smaller units that have been, or are being developed in parallel. We also provide a rationale for identifying which units are most suitably developed using systematic approaches versus those that are based on canonical knowledge for which an extensive literature review and evaluation is neither necessary nor practical.

## THE AOP CONCEPT IN BRIEF

The AOP concept seeks to pragmatically describe a chain of events from an initial interaction with a stressor through to an adverse health outcome in an intact organism. It builds on similar principles as the mode-of-action analysis used to assess the human relevance of animal toxicity data (Boobis *et al.*, 2008; Villeneuve *et al.*, 2014a). The AOP framework organizes biological knowledge into modular descriptions assembled from smaller building blocks, or units, of knowledge. The smallest unit is a key event (KE) that describes some measurable change in a biological system (an observation). Pairs of KEs are linked together into upstream-downstream (cause-effect) pairs by key event relationships (KERs), which are assemblies of established biological knowledge and empirical evidence that connect the 2 KEs together.

An individual AOP is composed of a unique sequence of KEs and KERs that connect the initial interaction between the stressor and the organism. Typically, they do so at the molecular level (termed molecular initiating event; MIE) to a consequent adverse effect at a level of the biological organization considered relevant to risk assessment, regulatory decision-making, or management of environmental or human health (termed the adverse outcome [AO]; OECD, 2018). AOPs do not aim to faithfully depict the complex molecular and cellular events taking place in the living organisms in their entirety (Draskau *et al.*, 2020). Rather, AOPs are stripped-down versions of toxicity pathways focusing on a number of essential events that serve as milestones or landmarks along the progression toward an AO (Ankley *et al.*, 2010; Villeneuve *et al.*, 2014a,b). In this context, it is important to recognize that the number of KEs in an AOP should ideally be limited to those KEs that are both essential and measurable, and for which evidence supports plausibility and potential predictive utility (OECD, 2018). Vitally, AOP development focuses on providing evidence of causality between upstream events and downstream effects, in providing a stressor agnostic mode-of-action analysis that supports inference or extrapolation across levels of biological organization. It is this evidence of causal relationships between events that provides the AOPs their value in supporting the application and interpretation of data from nonanimal test methods.

With this strong focus on establishing causal links between initial stressor interactions with biomolecules through to adverse effects, the KERs are, to a large degree, the most important modules of any robust AOP. The KERs are what provide the causal linkages for the progression down any given AOP to culminate in an AO. Thus, when developing an AOP, the largest evidence burden should be placed on the KERs. The KEs, which describe a measurable change in a biological state, are comparably much simpler to develop and document than KERs. They only require a minimum amount of general description; enough so that it is clear what a KE represents and how to measure it, but not so much that it becomes too specific for individual AOPs; eg, AOP-wiki KE-26 “Antagonism of Androgen receptor” or KE-1800 “Reduced granulosa cell proliferation of gonadotropin-independent follicles.” The idea is that KEs should be simple building blocks that can be shared between AOPs, even distant ones with regard to biological application domains. The question then becomes how much evidence is required to establish a KER.

Currently, there is no absolute rule for how to elaborate a KER, although systematic literature search approaches are encouraged. By this, we do not necessarily restrict the term “systematic review” to PRISMA guidelines (Moher *et al.*, 2009), but rather by searching literature using systematic search terms and, most importantly, providing a transparent description of how the literature was searched and selected. We also support this view, as systematic literature search approaches improve transparency, efficiency, and reuse in the assembly and documentation of the underpinning evidence, thus providing a higher level of scientific completeness and a stronger overall weight of evidence. Ultimately, the adoption of systematic approaches in AOP development will increase the broader acceptance of AOPs for chemical safety assessments; however, employing and documenting a systematic literature search and associated evidence evaluation is also a major hurdle with regard to fast-tracking AOP development, as it requires a substantial investment of time and resources. Therefore, we advocate for the adoption of a more pragmatic approach where only those KERs pertaining to cause-effect relationships that are not widely established and accepted are elaborated using systematic literature review approaches. Other KERs where the evidence is considered common knowledge, or canonical, should not require the same approach but rather rely on existing syntheses of knowledge through citation of a few key review articles or textbooks. Indeed, the OECD’s Users’ Handbook Supplement to the Guidance Document for Developing and Assessing AOPs says as much (page 40):

…it is recognised that there may be cases where the biological relationship between two KEs is very well established, to the extent that it is widely accepted and consistently supported by so much literature that it is unnecessary and impractical to cite the relevant primary literature. Citation of review articles or other secondary sources, like text books, may be reasonable in such cases. The primary intent is to provide scientifically credible support for the structural and/or functional relationship between the pair of KEs if one is known. (OECD, 2018)

Having said this, we appreciate the challenge in determining when a KER should be considered canonical or not and there will be many instances where this is difficult to agree upon. It is beyond the scope of this paper to provide a thorough guidance to this, but suffice to say, it will in the immediate future depend on agreements between developers and reviewers, all of which are experts in their respective fields.

## A PRAGMATIC APPROACH TO AOP DEVELOPMENT

To facilitate more rapid development and endorsement of AOPs under the OECD program, we propose 2 ways of streamlining the process. The first is to allow for the separate scientific review of a smaller unit of knowledge aggregation in the AOP landscape, namely the KER, preferably associated with an intended full AOP or AOP network (AOP networks are clusters of AOPs sharing as a minimum one KE, that like AOPs, can be considered collections of KERs). The second is to only incorporate extensive systematic literature review approaches for KERs that are not considered canonical knowledge in the field.

###  

#### The KER as a Pragmatic Unit of Development and Evaluation

In the early conception phases of the AOP framework, it was perceived that a single unbranched, linear AOP would be the most practical unit of development and evaluation (Villeneuve *et al.*, 2014a). The authors contended that restricting development to a single linear chain of KEs and KERs, from one MIE to one AO, would reduce inconsistencies in AOP descriptions between developers and simplify the evaluation of predictive relationships. However, with the integration of a more formal evidence assembly and evaluation process into the framework (Becker *et al.*, 2015), and subsequent additions of sections to the KER description templates to capture both evidence for causality as well as more detailed quantitative understanding of the KER (Wittwehr *et al.*, 2017), the task of KER description has expanded beyond the relatively concise and *ad hoc* approach of early AOP development. In addition, as AOPs began to be subjected to scientific review and endorsement, there has been increasing demand to incorporate certain elements of systematic literature review as a means to mitigate the tendency for confirmation bias when assembling evidence from the literature. Consequently, the evidence requirements for establishing such predictive relationships are more burdensome than initially anticipated (Carusi *et al.*, 2018). The average time to develop a single AOP according to OECD guidance, from the time it is first created in the AOP-wiki to the time it is reviewed and endorsed, is in excess of 3 years. Clearly, this is a challenge.

Furthermore, a disconcerting number of AOPs in the wiki remain “under development” and have remained largely incomplete for long periods of time (effectively abandoned by their authors). In fact, and as mentioned previously, since the launch of the AOP-wiki platform in 2014 only 17 of the 335 AOPs currently described in the wiki have completed formal peer review and endorsement cycle established by the OECD. Although there are many factors responsible for the slower-than-anticipated growth of the AOP-KB, we contend a smaller unit of development and review than an AOP would be more pragmatic and tractable for many contributors and would likely lead to both more participation in AOP development and higher-quality contributions.

**Figure 1.**
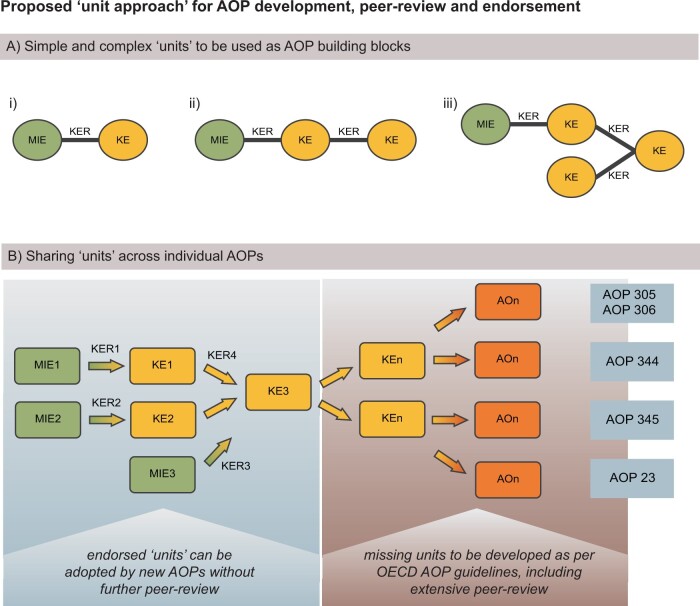
Approach to develop, endorse and reuse smaller AOP units for the AOP-KB. A, As much as KEs are the modular building blocks of the AOP universe, KERs represent the causal knowledge of biological events, and are thus the most critical components of any AOP or AOP network. The smallest unit that is of any real value for regulatory purposes is 2 KEs connected by one KER (i), the second smallest is 3 KEs linked by 2 KERs (ii), with ever-increasing complexity as more KEs and KERs are added in a linear or branched manner (iii). B, With fully developed, peer reviewed and endorsed units that themselves are smaller than an AOP, the development of new AOPs will be fast-tracked by way of adopting and reusing these units when developing new AOPs. As exemplified, a small network of well-established biological pathways (left box) could form the basis for numerous new AOPs (right box; here exemplified by actual AOP-wiki entries pertaining to perturbed androgen signaling). Abbreviations: AOP, adverse outcome pathway; KB, knowledge base; KE, key event; KER, key event relationship.

To reiterate, AOPs are comprised of sequences of KEs connected by KERs (Figure 1). Although KEs can inform what assays and endpoints to focus on to verify that a stressor is impacting an organism in an expected manner, the KEs in isolation have no value for inference. However, when pairs of KEs are linked by KERs summarizing the causal functional relationship, the interconnected entities become a very strong unit to support inference. Establishing KERs can, in many instances, require extensive literature reviews and/or generation of novel experimental data followed by robust peer review by experts to be generally accepted. Because of the causal relationships they infer, KERs have the greatest requirement for evidence among the various modular units of the framework. Indeed, KERs are frequently described as “the unit of inference or extrapolation” within an AOP (Villeneuve *et al.*, 2014a). Furthermore, a result of the modular nature of the AOP framework is that such KERs are frequently reused across multiple AOPs. Thus, a single KER could potentially be involved in the assessment of many diverse stressors. It is therefore worthwhile to direct substantial resources into their individual development to ensure their scientific quality. We therefore argue that the KER “unit” should be formally recognized as the core building block, or unit of knowledge assembly, within the AOP-KB, following independent review.

Equally important to AOP development are the resources required for their peer review. Because AOPs typically span from the molecular level up to individual and even population-scale effects, many reviewers do not have the requisite background or expertise to review all the KERs that compose an AOP. Furthermore, when individual KER descriptions are many pages in length and integrate multiple lines of evidence from dozens of primary sources, review of the content of multiple KER descriptions can be both a daunting and time-consuming task. In addition, given the modular nature of the framework, KERs that are shared by more than one AOP may be subjected to multiple peer reviews. This is not an efficient use of the time and effort that subject matter experts volunteer to ensure the quality of the program. Instead, we advocate that high-quality KERs should be subject to one single high-quality review. Once reviewed, such units could be adopted as preapproved units to be incorporated into more elaborate AOPs or AOP networks. Through this approach, KERs that are formally peer reviewed provide future developers and peer reviewers with ready-to-use units that will lessen the burden for AOP developments going forward.

Recognizing that KER descriptions (like all elements of AOPs) are living documents (Villeneuve *et al.*, 2014a), their content may continue to evolve. History tracking within the AOP-wiki can make it a simple matter to identify content that has been updated or added since the KER was last reviewed, thereby greatly reducing the overall review burden when a previously reviewed KER is added to a new AOP.

In our view, focusing development and review resources on individual high-quality KERs as a primary unit of contribution, instead of full AOPs, would serve 3 purposes:

1. It will encourage more experts to embark on AOP development activities, as they are no longer faced with the daunting task of completing an entire AOP before review and endorsement, or assembling and coordinating a team with the breadth of knowledge and expertise to span all levels of biological organization.
2. It will fast-track endorsed entries into the AOP-wiki platform, as the units will be faster to construct than full AOPs. Likewise, scientific reviews would be narrower in scope, requiring less time to review and enabling better targeting of appropriate subject matter experts, thereby improving quality.
3. It would make more efficient use of reviewers’ time, as reviewer hours are not spent reviewing previously endorsed content in AOP-wiki, thereby combating reviewer fatigue and disenchantment.

#### Literature Review Approaches

In addition to shifting the focus to KERs as the primary unit of development and review, another pragmatic approach to AOP development is to be selective about when a systematic literature review is needed, and when it is not. Although systematic review approaches are recognized to be more objective and transparent than narrative review approaches, they can require a tremendous amount of effort, screening, and evaluation of tens, hundreds, or even thousands of papers by multiple experts. With respect to AOP development, the adoption of some aspects of systematic review approaches is encouraged, but only to the extent that they add confidence that the authors have not cherry-picked literature to support a preconceived hypothesis; ie, not used a biased approach to promote a viewpoint potentially disputed by alternative evidence. Thus, authors should be encouraged to employ systematic review approaches where it is most critical to establishing scientific confidence in a more uncertain aspect of an AOP, but should also recognize where such measures are unnecessary to generate confidence. In essence, we suggest using different approaches depending on the KERs in question.

When the AOP includes KERs, or bigger units, that are considered canonical (“textbook”) knowledge, it should suffice to rely on leading review articles or similar from the open literature rather than employing systematic review approaches. Likewise, if the evidence available in the literature is sparse or lacking altogether for the KER in question, it may be adequate to simply cite peer-reviewed experimental results that offer the only available evidence pertaining to a relationship, such as a targeted experiment designed by the contributor(s) themselves. It is only in cases where there is extensive evidence already in the literature, but that evidence is not widely known and/or broadly accepted as canonical knowledge, that a systematic review-like approach would be appropriate. Thus, a critical element to describing the KER should be providing clear explanation for the approach taken to assembling the evidence and the rationale for that approach.

## AN EXAMPLE OF PRAGMATIC AOP DEVELOPMENT

**Figure 2.**
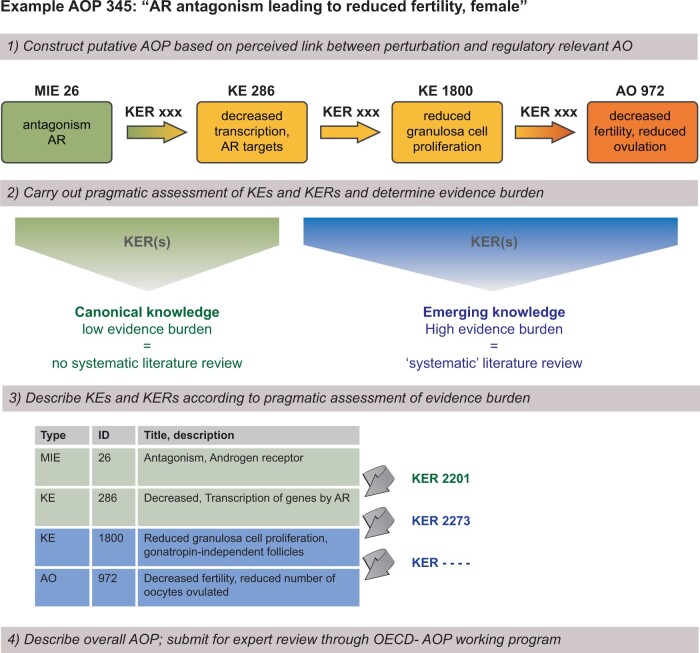
Developing AOPs by adopting pragmatic approaches to elaborating KERs. AOPs comprise a series of KEs from an MIE through to an AO that are linked by KERs. Although the KEs are important building blocks, KERs represent the most elaborate and important information for any AOP to be of regulatory use as it provides the causal link between chemical perturbation and adverse effects in intact organisms. Taken together, all KEs and KERs that form an AOP make up a substantial body of supporting knowledge, with a single KER easily comprising a large database of articles that itself could fill an extensive review article in a scientific journal. To lessen the burden on both developers and reviewers of AOPs under development, KERs should be substantiated differently depending on the level of general acceptance of the causal relationship in question. In instances where the knowledge is considered “text-book,” or canonical, there should be no need for an extensive review process, but rather a reliance on pre-existing literature reviews. When the knowledge is not considered canonical, systematic literature approach should be adopted before being subjected to peer review and ultimately endorsement. Abbreviations: AO, adverse outcome; AOP, adverse outcome pathway; KE, key event; KER, key event relationship; MIE, molecular initiating event.

To illustrate what pragmatic AOP development would look like in practice, we provide the example of AOP 345, which we are currently developing. AOP 345 links androgen receptor antagonism to reduced fertility in females (Figure 2; https://aopwiki.org/aops/345; last accessed September 10, 2021).

In this example, we reused existing KERs from separate AOPs that describe AOs of androgen receptor antagonism in male offspring to establish the upstream events of AOP 345. Because the first steps in describing androgen receptor antagonism (MIE 26) leading to altered transcription of genes by the androgen receptor (KE 286; KER 2201; https://aopwiki.org/relationships/2201; last accessed September 15, 2021) are also directly relevant for AOP 345, we were able to adopt this “unit” as is. Had this unit already been peer reviewed and endorsed, the remaining focus could have been placed on the downstream events only. Incidentally, this also illustrates how we envision AOP networks will naturally develop within the AOP-wiki framework as more AOPs or AOP units are added (Figure 1B).

To the point of a pragmatically selective application of systematic review approaches, which can be adopted by any AOP from any field, the first KER unit of AOP 345 represents a causal relationship between an MIE and a KE that is regarded as canonical.androgen receptor is a ligand-regulated nuclear receptor that regulates transcription in response to androgens (testosterone and dihydrotestosterone) that bind to the receptor (Brinkmann *et al.*, 1999; Claessens *et al.*, 2008). So if androgen binding is prevented for instance by antagonistic molecules, the androgen receptor cannot carry out its function: bind DNA to regulate gene transcription. Because experts in the field would most likely not argue against this “factual,” functional relationship, it should suffice to use a more “narrative review approach” when populating this unit on AOP-wiki.

With regard to the downstream events, on the other hand, the story is quite different. Here, we are proposing a causal relationship between 2 KEs (altered transcription by androge nreceptor and reduced proliferation of granulosa cells) that has support from the literature, but the evidence is either limited or there are conflicting reports. In this instance, a more systematic review approach should be adopted to avoid bias in the supporting evidence, as well as making sure relevant literature is included and that the process of literature selection is transparent for the end-users of the AOP.

There is an ongoing debate about how to conduct literature searches for AOP development. In our view, systematic approaches should be used, but only where appropriate, as discussed above. Furthermore, it is our opinion that the systematic literature approach does not need to follow the PRISMA guideline (http://www.prisma-statement.org/; last accessed June 12, 2021), but rather an approach more aligned with the weight-of-evidence approach used for chemical safety assessments. Undoubtedly, this is a debate that will continue beyond this article, yet hopefully, result in a general “best practice” framework within the near future. Regardless, the most critical points to consider when developing noncanonical KERs is to include descriptions of the strategies that were employed for literature searches, including inclusion and exclusion criteria. Of course, such criteria should be nonbiased and follow appropriate weight-of-evidence approaches to reach an overall conclusion as to the strength of the KER. Most importantly, the strategies employed should be transparent and accessible, allowing for scrutiny by external assessors and users and be updated as appropriate when new information becomes available.

## SUMMARY AND CONCLUSIONS

The AOP framework holds great promise for providing chemical risk assessors and the regulatory community more broadly with an easily accessible repository of causal relationships needed to provide plausibility links between molecular perturbations and AOs that are relevant for human and environmental health. Although there has been an increase in AOP development activities across many toxicological disciplines over the past decade, the number of AOPs that have been developed up to a level where they can be effectively used in a risk assessment or regulatory context remains relatively limited. A major hurdle in AOP development is the amount of time and effort that is required for the detailed description of all of the KERs involved in the AOP, including an assessment of the weight of evidence, the quantitative understanding, and so forth. This is not to say that it represents the only hurdle to be tackled to better streamline AOP development and a broader adoption for regulatory purposes. Other challenges that need to be addressed include the adoption of more standardized ontologies (Wang, 2020), machine readability of content with the AOP-KB, potential copyright issues related to journal publications of open-source AOP content, and more user-friendly guidance documents for the application of AOPs. But most importantly at present, the biggest hurdle for AOP usability lies in lack of content.

To address this last point, we propose with this paper to adopt a pragmatic AOP development and evaluation approach that focuses on individual KERs as the primary unit for assembling and reviewing information. In addition, we propose to selectively apply systematic-like literature review approaches only to those KERs for which no canonical knowledge supporting the causal relationship described in the KER exists. To ensure that individually developed KERs remain connected to a complete AOP, we suggest providing a blue-print AOP diagram as a placeholder to provide the relevant broader context. Table 1 provides an overview of the different steps of this suggested approach.

**Table 1.** A Pragmatic Approach to AOP Development and Evaluation

1. Development	
1.1. Draw AOP blue-print diagram	Outline relevant AOP(s) and connect KEs by KERs to provide context and structure Identify and describe relevant KEs Identify canonical KERs versus KERs that require in-depth development
1.2. Develop individual KER	If canonical: rely on leading review articles from the open literature Use systematic review approaches for KER development when required
2. Evaluation and endorsement	
2.1. Scientific review of KER	As a “KER Report” journal article similar to the existing “AOP Report” article format At the OECD EAGMST level
2.2.Endorsement of AOP	Assemble complete AOP or AOP network, completely or in part based on KERs that have already been reviewed OECD (WPHA/WNT) review and endorsement of entire AOP (network)

Abbreviations: AOP, adverse outcome pathway; KE, key event; KER, key event relationship; OECD, Organisation for Economic Co-operation and Development; EAGMST, Extended Advisory Group on Molecular Screening and Toxicogenomics; WPHA, Working Party on Hazard Assessment; WNT, Working Group of National Coordinators of the Test Guidelines program.

## DECLARATION OF CONFLICTING INTERESTS 

The authors declare that they have no direct conflicts of interest pertaining to the work presented in this manuscript. The contents of this manuscript neither constitute nor necessarily reflect US EPA policy. Mention of trade names or commercial products does not constitute endorsement or recommendation for use. The European Union cannot be held responsible for any use that may be made of the information contained in this paper.

## FUNDING 

This work received funding from the Danish Environmental Protection Agency as a project under the Centre on Endocrine Disrupters (CeHoS); the Swedish Chemicals Agency and the Swedish Research Council for Sustainable Development FORMAS (grant number 2020-01621); the European Union’s Horizon 2020 FREIA (Duursen et al, 2020) under grant agreement No. 825100 (FREIA). The University of Antwerpen received funding from the European Union’s Horizon 2020 project ERGO (Holbech et al, 2020) under grant agreement No. 825753.
